# Cytotoxic Cembranes from Indonesian Specimens of the Soft Coral *Nephthea* sp

**DOI:** 10.3390/md8072142

**Published:** 2010-07-13

**Authors:** Hedi Indra Januar, Ekowati Chasanah, Cherie A. Motti, Dianne M. Tapiolas, Catherine H. Liptrot, Anthony D. Wright

**Affiliations:** 1 Indonesia Research Center for Marine and Fisheries Product Processing and Biotechnology, Jl. KS Tubun Petamburan VI, Jakarta 10260, Indonesia; E-Mails: idjanuar@gmail.com (H.I.J.); ekowati_ch@yahoo.com (E.C.); 2 Australian Institute of Marine Science, PMB No. 3, Townsville MC, Townsville, 4810, Australia; E-Mails: c.motti@aims.gov.au (C.A.M.); d.tapiolas@aims.gov.au (D.M.T.); 3 University of Hawaii, College of Pharmacy, 34 Rainbow Drive, Hilo, HI 96720, USA

**Keywords:** Indonesia, marine natural products, soft coral, Nephthea sp., anticancer, NMR, cembrane

## Abstract

Methanol extracts of two specimens of the soft coral *Nephthea* sp. collected from the Seribu Islands, Indonesia, were active in an anticancer bioassay. One new (**1**) and four known diterpenes (**2**–**5**) based on the cembrane carbon skeleton were isolated from these extracts, as was arachidonic acid (**8**). The structures of all compounds were elucidated using NMR, including 1,1-ADEQUATE and 1D gradient selective NOESY where applicable to determine the relative stereochemistry. Spectroscopic data, including ^1^H and ^13^C NMR, UV, IR and optical rotations are reported when enough material was available and where this has not been done previously. Inhibition assays employing three cancer cell lines; SF-268 (CNS), MCF-7 (breast), and H460 (lung) were used to guide the isolation of all compounds.

## 1. Introduction

A large diversity of marine organisms have been shown to produce secondary metabolites as a means of defense [1–4], many of these compounds also possess interesting biological activities [5–7]. Soft corals are no exception [8–12]. Investigations of soft corals from Indonesian waters have been limited with only six such reports on six unrelated soft coral species [13–18], the first of these appearing in 1997 [13]. Since 2002 [15,16], aside from the research undertaken by Fattorusso *et al.* [17], Wang *et al.* [18], and the current authors [19–22], there is little work being done with soft corals from this region of the world, even though they are likely to be rich sources of biologically active secondary metabolites. The information presented here resulted from a formal cooperation between the Indonesia Research Center for Marine and Fisheries Product Processing and Biotechnology, and the Australian Institute of Marine Science (AIMS), funded by an AusAID PSLP Indonesia grant. The investigation of two *Nephthea* (Alcyonacea, Nephtheidae) species, whose methanol (MeOH) extracts exhibited anticancer properties, resulted in the isolation of a new cembrane 3,4-epoxy-nephthenol acetate (**1**) along with five known compounds: decaryiol (**2**), 15-hydroxy-cembrenene (**3**), 2-hydroxy-nephthenol (**4**), nephthenol (**5**) and arachidonic acid (**8**). This report describes the structural elucidation of (**1**) and clearly shows that soft corals of Indonesian origin have significant potential as sources of biologically active and drug development lead compounds.

## 2. Results and Discussion

Compound **1** was isolated as a yellow oil from *Nephthea* sp. specimen A. Mass spectrometric analysis of the compound showed it to have the molecular formula C_22_H_36_O_3_ and therefore have five double bond equivalents of unsaturation. From the ^1^H and ^13^C NMR data of **1**, it was evident that the molecule contained two C=C double bonds (δ_C_ 123.6 d, 126.2 d, 132.4 s, 134.9 s) and one C=O (δ_C_ 170.2 s) double bond as the only multiple bonds establishing it as bicyclic. The 1D and 2D NMR data of **1** also revealed the presence of an acetate (δ_C_ 170.2 s, 85.6 s, 22.8 q, 1.98 s [C-21, 22 and 15, respectively]) and an ether (δ_C_ 63.4 d and 61.5 s, δ_H_ 2.84 [dd, 9.4, 3.4 Hz], [C-3 and C-4, respectively]). From the ^1^H-^1^H COSY spectrum of **1**, three continuous chains of coupling were discerned; from H_2_-13 to H-3 via H_2_-14, H-1 and H_2_-2, respectively; from H_2_-5 to H_3_-19, via H_2_-6 and H-7; and from H_2_-9 to H_3_-20, via H_2_-10, and H-11, respectively. From long-range ^1^H-^13^C couplings observed between H_3_-18 and C-3, C-4 and C-5; between H_3_-19 and C-7, C-8 and C-9; and between H_3_-20 and C-11, C-12 and C-13, and from 1,1-ADEQUATE cross-peaks [23] (See Table 1), it was possible to link together the three proton spin systems into a continuous carbon chain to form the first ring within **1**. The chemical shifts associated with C-3, C-4 and H-3 indicated the ether functionality was in fact an epoxide, and hence formed the second ring within **1**, fulfilling the requirement for five double bond equivalents of unsaturation. The protons associated with two of the three unassigned methyl groups demonstrated long-range ^1^H-^13^C couplings between each others carbon and, C-1 and C-15, giving rise to a *gem*-dimethyl constellation attached to C-1, leaving the acetate function to reside at C-15, leading to the planar structure of **1**. The geometry of the two C=C double bonds within **1** were both deduced to be *E* based on the ^13^C NMR chemical shifts of C-19 and C-20 (δ_C_ 16.7 and 15.3, respectively). Based on the observation that the ^13^C NMR signals for C-2 and C-18 (δ_C_ 30.8 and 16.6, respectively) occurred significantly upfield of that for C-5 (δ_C_ 39.3), resulting from steric compression due to their *cis* orientation, the relative stereochemistry of the epoxide is as shown in **1** [24]. This deduction was further supported by comparison of the ^13^C NMR chemical shift data for C-3, C-4 and C-18 in **1** with those for **6**, as well as the ^1^H NMR data associated with H-3 in both compounds. Selective 1D NOESY excitation of the epoxide proton H-3 (δ 2.84) gave rise to signals corresponding to H-1 (δ 2.22), H-2 (δ 1.56), H-5 (δ 1.13), H-7 (δ 5.23) and H-11 (δ 5.13); this information, and comparison of both ^1^H and ^13^C NMR data with those for **6** confirmed the relative configuration at C-1, 3 and 4 to be deduced as shown in **1**. Unfortunately, the absolute stereochemistry of **1** could not be determined as the compound was unstable and degraded before an optical rotation could be obtained. Literature searches for this molecule revealed it to be a new compound and the acetylated derivative of 3,4-epoxy-nephthenol, **6** [28].

HRESIMS of **2**, also isolated from *Nephthea* sp. specimen A, resulted in an [M + Na]^+^ ion corresponding to a molecular formula of C_20_H_34_O_2_ and hence four degrees of double bond unsaturation. The ^1^H and ^13^C NMR data of **2** revealed the presence of two C=C double bonds (δ_C_ 132.6 s, 132.1 s, 128.0 d and 127.7 d) as the only multiple bonds within the molecule indicating a bicyclic structure. Carbon chemical shifts indicated the presence of three oxygenated carbons (δ_C_ 76.8 s, 75.1 s and 70.3 d), two of which formed an ether linkage accounting for one of the rings within **2**, and the third an alcohol (δ_H_ 4.20 [dd, 11.7, 5.6 Hz, axial proton]). Database and literature searches using this information and comparison of spectroscopic data with literature values confirmed **2** to be the known compound decaryiol [28]. Selective 1D NOESY experiments enabled the relative configuration of **2** to be determined as shown; NOESY correlations were observed from H-3 to H-1, H-2, H-5, H-6, H-7 and H-11; from H-7 to H-3, H-5, H-6 (δ_H_ 2.62 br and 1.88 s) and H-9 (δ_H_ 2.20 br and 2.17 s); from H-11 to H-1, H-2, H-3, H-9, H-10 and H-13; and from H-18 to H-2.

**Figure 1:**
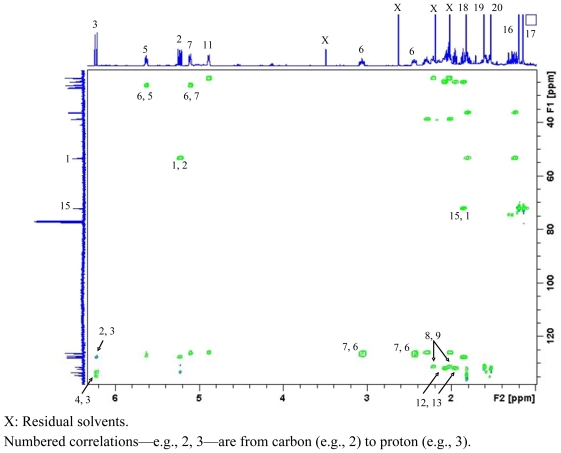
1,1-ADEQUATE spectrum of **3** (600 MHz basic frequency, CDCl_3_). X: Residual solvents. Numbered correlations—e.g., 2, 3—are from carbon (e.g., 2) to proton (e.g., 3).

Accurate mass measurement of **3** showed it to have the molecular formula C_20_H_32_O and to contain five double bond equivalents of unsaturation. From the ^1^H and ^13^C NMR data of **3** it was evident that the molecule contained four C=C double bonds (δ_C_ 134.6 s, 133.4 d, 132.1 s, 131.5 s, 127.9 d, 127.3 d, 126.3 d and 126.2 d), revealing it to be monocyclic. It was also evident from this data that **3** had one carbon attached to an oxygen, therefore an hydroxyl, functionality (δ_C_ 72.3 s). As for **1**, three chains of ^1^H-^1^H coupling were discerned from the COSY spectrum of **3**; from H_2_-13 to H-3 via H_2_-14, H-1 and H_2_-2, respectively; from H_3_-18 to H_3_-19, via H-5, H_2_-6 and H-7; and from H_2_-9 to H_3_-20, via H_2_-10, and H-11, respectively. From long-range ^1^H-^13^C couplings observed between H_3_-18 (δ_C_ 19.9, Δ^4^ *Z* configuration) and C-4 and C-5; between H_3_-19 (δ_C_ 14.5, Δ^7^ *E* configuration) and C-7, C-8 and C-9; and between H_3_-20 (δ_C_ 14.3, Δ^11^ *E* configuration) and C-11, C-12 and C-13, and 1,1-ADEQUATE [23] cross-peaks (Figure 1), it was possible to link together the three proton spin systems into a continuous carbon chain to form the one ring within **3**. HMBC and COSY correlations also confirmed two of the C=C double bonds (Δ^2^ and Δ^4^) were conjugated; H-2 to C-4, H-3 to C-4 and C-5, and H-5 to C-3, and that Δ^2^ had *E* configuration (H-2: δ_H_ 5.20 [dd, 15.3, 10.0 Hz]; H-3: δ_H_ 6.20 [d, 15.3 Hz]). Comparison of these data with literature values for cembrenene (**7**) previously isolated from *Sinularia mayi* [26], (Table 2) indicated the structures to be similar. The protons associated with the two remaining methyl groups CH_3_-16 and CH_3_-17 demonstrated long-range ^1^H-^13^C couplings to each others protons and carbon and to C-1 and C-15 giving rise to a *gem*-dimethyl constellation attached to C-1 with the hydroxyl moiety residing at C-15, and the planar structure as shown in **3**, 15-hydroxy-cembrenene. Literature searches for this molecule yielded a report detailing the synthetic dehydration of 2-hydroxy-nepthenol to give **3** [27]. The current report, however, is the first time **3** has been isolated from a natural source.

A second *Nephthea* sp., specimen B, was investigated for its anticancer activity with one of the active fractions yielding **4**, having the same molecular formula as **2**, C_20_H_34_O_2_. Comparison of the ^1^H and ^13^C NMR data of **4** with those of **2** showed it to contain only two oxygenated carbons (δ_C_ 71.1 d and 74.6 s) as compared to the three in **2**, and three C=C double bonds (δ_C_ 139.5 s, 135.4 s, 133.3 s, 127.9 d, 124.9 d and 123.9 d) rather than two as found in **2**, confirming it to be a monocyclic diol. Its 1D and 2D NMR data confirmed it to be 2-hydroxy-nephthenol [27]. After leaving **4** to stand for one week in CDCl_3_, it was found to have quantitatively rearranged to **3**. Given this result, it is unclear whether **3**, previously reported synthetically [27] and reported here from *Nephthea* sp. specimen A, was in fact a natural product, or a by-product of the isolation process [27]. However, close inspection of fractions from specimen A shortly after they were prepared did not reveal the presence of any **4**, leading us to believe that **3** is in actual fact naturally occurring.

A second active compound, **5**, was isolated from specimen B. The mass spectrum of **5** showed an [M + Na]^+^ ion in its HRESIMS consistent with the molecular formula C_20_H_34_O and four degrees of C=C unsaturation. The ^1^H and ^13^C NMR data of **5** showed it to contain three C=C double bonds (δ_C_ 134.1 s, 133.4 s, 133.0 s, 125.9 d, 125.8 d and 125.0 d) as well as one hydroxyl group (δ_C_ 75.1 s), making it a monocyclic alcohol. Comparison of its spectroscopic data with literature values confirmed **5** to be nephthenol [28].

Compound **8** was isolated as a yellow oil with the molecular formula of C_20_H_32_O_2_, as determined by HRESIMS measurement of its [M – H]^−^ ion. Analysis of the ^1^H and ^13^C NMR spectral data of **8** in CDCl_3_ revealed signals consistent with the presence of a carboxyl group (δ_C_ 177.9 s) and eight sp^2^ methine carbons (δ_C_ 130.5 d, 129.0 d, 128.8 d, 128.6 d, 128.3 d, 128.1 d, 127.9 d and 127.5 d) accounting for all five of the C=C double bond equivalents of unsaturation within the molecule and showing it to be acyclic. Signals from three methylene carbons adjacent to *cis* double bonds were observed at δ_C_ 25.6, 25.6 and 25.6, as well as for seven other methylene carbons and one methyl group (δ_C_ 14.0). Comparison of these values with literature values confirmed **8** as the fatty acid arachidonic (20:4n-6) acid [29].

Compounds **1**–**5**, and **8** were screened for their whole cell anticancer activity against three human tumor cell lines (SF-268 [CNS], MCF-7 [breast], H460 [lung]). All compounds demonstrated weak (GI_50_ > 100 μM) non-selective activity towards the three cell lines.

## 3. Experimental Section

### 3.1. General experimental

C18 flash vacuum chromatography was performed using Phenomenex C18 (50 μm). HPLC was performed employing a Phenomenex Luna C18 column (250 × 21 mm) attached to a Shimadzu HPLC system consisting of a Shimadzu SCL-10Avp system controller equipped with a Shimadzu LC-10AT pump, Shimadzu SPD-M10Avp photodiode array detector, Shimadzu FRC-10A fraction collector and Shimadzu SIL-10A auto sampler using Shimadzu Class-VP software. IR spectra were measured on a Nicolet Nexus FTIR. Optical rotations were collected on a Jasco 715 CD polarimeter. All NMR spectra were recorded on either a Bruker Avance 600 MHz NMR spectrometer complete with cryoprobe, or a Bruker Avance 300 MHz NMR spectrometer, with spectra referenced to residual ^1^H and ^13^C resonances in the deuterated solvents. Accurate mass spectrometric data were measured using a Bruker BioApex 47 FT mass spectrometer. All other details as previously published [30].

### 3.2. Animal material

*Nephthea* sp. specimen A was collected from Seribu Islands, DKI Jakarta, Indonesia, at a depth of 10 m, at 10:21 am, on the 22 July 2005; *Nephthea* sp. specimen B was collected from Seribu Islands, DKI Jakarta, Indonesia, at a depth of 15 m, at 1:10 pm, on the 22 June, 2005. Soft coral taxonomy was undertaken by K. Fabricius, AIMS. A voucher sample for each specimen has been lodged with the Indonesia Research Center for Marine and Fisheries Product Processing and Biotechnology, Jakarta, Indonesia.

### 3.3. Bioassay

Natural product samples were assayed against three cell lines; SF-268, MCF-7 and H460 cells, as described in a previous study [31]. In brief, natural product samples, solubilized in DMSO and serially diluted in RPMI 1640 medium, were added to SF-268, MCF-7 and H460 cells so that the final doses ranged from 1000 μg/mL to 1 μg/mL. Total cellular protein was measured using the sulforhodamine B (SRB) assay as an indicator of cell number. Inhibition of growth by 50% (GI_50_) was determined by comparing the sample treated values to those of vehicle only control and time 0 readings.

### 3.4. Extraction and isolation

Extract A: The organic solubles (0.84 g), obtained by employing repeated extraction of 100.00 g wet weight of *Nephthea* sp. specimen A with MeOH, were filtered through a plug of reversed phase C18 silica using MeOH as eluent. The MeOH was removed under reduced pressure and the resultant dry extract subjected to preparative RP-HPLC (9 mL/min, gradient elution from 15% MeCN:H_2_O to 100% MeCN; column 250 × 20 mm RP Luna C18 (2), Phenomenex, over 70 mins) to yield 63 fractions. Three of the 63 fractions, 27, 30 and 32, were found to be active in the applied bioassay systems. ^1^H NMR analysis of these fractions showed them to be a 1:1 mixture of **3** and **4** (10.0 mg, 1.19% organic extract), **8** (10.0 mg, 1.19% organic extract), and **5** (10.0 mg, 1.19% organic extract), respectively.

Extract B: The organic solubles (3.31 g), obtained by employing repeated extraction of 300.00 g wet weight of *Nephthea* sp. specimen B with MeOH, were filtered through a plug of reversed phase C18 silica using MeOH and DCM as eluents. The MeOH and DCM were removed under reduced pressure and the resultant dry extracts (1.49 g and 0.10 g, respectively) subjected to preparative RP-HPLC. The MeOH extract (9 mL/min, gradient elution from 15% MeCN:H_2_O to 100% MeCN; column 250 × 20 mm RP Luna C18 (2), Phenomenex, over 70 mins) yielded 57 fractions of which only one, fraction 35, was found to be active in the applied bioassay systems. ^1^H NMR analysis of this fraction showed it to be **2** (57.9 mg, 1.75% organic extract). The DCM extract (9 mL/min, gradient elution from 15% MeCN:H_2_O to 100% MeCN; column 250 × 20 mm RP Luna C18 (2), Phenomenex, over 70 mins) yielded 55 fractions of which two, fractions 16 and 18, were found to be active in the applied bioassay systems. ^1^H NMR analysis of these fractions showed them to be **3** (2.3 mg, 0.07% organic extract), and **1** (0.8 mg, 0.02% organic extract), respectively.

*Compound* ***1*** *(3,4-Epoxy-nephthenol acetate)*. A yellow oil; [α]_D_ Sample decomposed prior to measurement; ^1^H (600 MHz, CDCl_3_), and ^13^C (150 MHz, CDCl_3_) NMR data see Table 1; HRESIMS *m/z* found 371.2563 for [M + Na]^+^ (calcd for C_22_H_36_O_3_Na 371.2557).

*Compound* ***2*** *(Decaryiol)*. A yellow oil [α]_D_ ^20^ + 27.2° (*c* 0.01), cf + 69.0° [25]; ^13^C (150 MHz, CDCl_3_) NMR data see Table 2; HRESIMS *m/z* found 329.2458 for [M – H]^+^ (calcd for C_20_H_34_O_2_Na 329.2451); and all remaining data as previously published [25].

*Compound* ***3*** *(15-Hydroxy-cembrenene)*. A yellow oil; [α]_D_ Sample decomposed prior to measurement; ^1^H (600 MHz, CDCl_3_) and ^13^C (150 MHz, CDCl_3_) NMR data see Table 2; HRESIMS *m/z* found 311.2330 for [M – H]^+^ (calcd for C_20_H_32_ONa 311.2345); and all remaining data as previously published [27].

*Compound* ***4*** *(2-Hydroxy-nephthenol)*. A clear oil ^13^C (150 MHz, CDCl_3_) NMR data see Table 2; HRESIMS *m/z* found 329.2452 for [M + Na]^+^ (calcd for C_20_H_34_O_2_Na 329.2451); and all remaining data as previously published [27].

*Compound* ***5*** *(Nephthenol)*. A clear oil; ^13^C (150 MHz, CDCl_3_) NMR data see Table 2; HRESIMS *m/z* found 313.2517 for [M – H]^+^ (calcd for C_20_H_34_ONa 313.2502); and all remaining data as previously published [28].

*Compound* ***8*** *(Arachidonic acid)*. A yellow oil; ^13^C (150 MHz, CDCl_3_) NMR data see Table 2; HRESIMS *m/z* found 303.2325 for [M – H_2_O + Na]^+^ (calcd for C_20_H_31_O_2_ 303.2330); and all remaining data as previously published [29].

## Figures and Tables

**Scheme 1:**
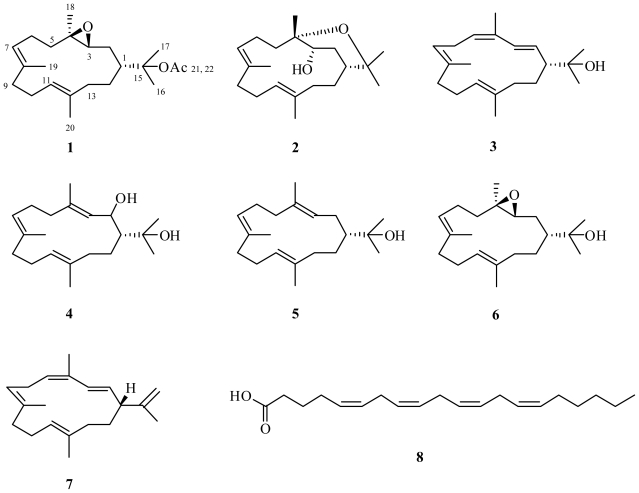
Structures of compounds **1**–**8** referred to throughout the publication.

**Table 1:** ^1^H and ^13^C NMR data (600 MHz basic frequency, CDCl_3_) for 3,4-epoxy-nephthenol acetate (**1**) and ^13^C NMR data for 3,4-epoxy-nephthenol (**6**) (22.63 MHz, CDCl_3_)

No.	δ_C_, mult.^a^(**1**)	δ_H_ (mult., *J* in Hz) (**1**)	COSY (**1**)	gHMBC (**1**) b	1,1-Adequate (**1**) b	δ_C_, mult. (**6**) ^25^
1	39.5 d	2.22 (m)	2, 14	2, 3, 14, 15, 16, 17	2, 14, 15	44.1 d
2	30.8 t	1.56 (m)	1, 3	3, 4, 14, 15		29.7 t
3	63.4 d	2.84 (dd, 9.4, 3.4) cf. 2.82 (dd, 9.2, 4.4)^25^	2	1, 2, 4, 5	2	63.0 d
4	61.5 s					61.9 s
5	39.3 t	2.06 (ddd, 13.3, 5.4, 2.9) 1.14 (dt, 13.3, 3.2)	5b, 6 5a, 6	4, 7 4, 6, 7, 18	4, 6	39.9 t
6	23.8 t	2.26 (m) 1.97 (m)	5, 6b, 7 6a, 7	4, 5, 7, 8 4, 5, 7, 8, 9	7 5	23.6 t
7	123.6 d	5.23 (brt, 7.6)	6a, 6b, 19	6, 9, 19	6, 7	125.9 d *
8	134.9 s					133.8 s *
9	39.8 t	2.03 (m) 2.18(m)		7, 8, 10, 11, 19	10 8, 10	36.6 t *
10	25.0 t	2.17 (m)	11	8, 9, 11	9, 11	24.9 t
11	126.2 d	5.13 (brt, 7.5)	10, 20	10, 13, 20	10, 11, 13	123.9 d *
12	132.4 s					134.9 s *
13	35.6 t	2.19 (m)	14a, 14b	11, 12	12, 14	39.1 t *
14	28.9 t	1.89 (m) 1.34 (m)	1, 13, 14b 1, 13, 14a	1, 2, 12, 13,15, 17 12, 15, 17	13	28.1 t
15	85.6 s					73.3 s
16	23.5 q	1.45 (s)		1, 15,17	15	26.6 q
17	23.4 q	1.43 (s)		1, 15, 16	15	28.6 q
18	16.7 q	1.31 (s)		3, 4, 5	4	16.9 q
19	15.3 q	1.62 (brs)	7	7, 8, 9	8	16.0 q *
20	15.8 q	1.58 (brs)	11	11, 12, 13	12	15.5 q *
21	170.2 s					
22	22.8 q	1.98 (s)		21	21	

* Based on the shifts found for **1**, these carbon resonances probably need reassignment.

a Correlations are from proton to carbon;

b Multiplicities determined by DEPT.

**Table 2:** ^1^H and ^13^C NMR data (125 MHz, CDCl_3_) for 15-hydroxy-cembrenene (**3**); ^13^C NMR data (125 MHz, CDCl_3_) for decaryiol (**2**), 2-hydroxy-nephthenol (**4**) and nephthenol (**5**); (22.6 MHz, CDCl_3_) for 3,4-epoxy-nephthenol (**6**), cembrenene (**7**) and arachidonic acid (**8**).

**No.**	**2**	**3**	**3**	**7**	**4**	**5**	**8**
	δ_C_, mult. a	δ_C_, mult. a	δ_H_, (mult., *J* Hz)	δ_C_, mult.	δ_C_, mult. a	δ_C_, mult. a	δ_C_, mult. a
1	39.9 d	53.5 d	1.85 (ddd, 2.5, 9.9, 12.3)	49.0 s	53.8 d	48.3 d	177.0 s
2	28.8 t	127.9 d	5.21 (dd, 9.9, 15.5)	130.5d	71.1 d	28.4 t	33.0 t
3	70.3 d	133.4 d	6.21 (brd, 15.5)	132.3d	127.9 d	126.0 d	24.5 t
4	76.8 q	134.6 s		135.1 s	139.5 s	134.1 s	26.4 t
5	37.9 t	127.3 d	5.61 (brdd, 6.9, 9.0)	126.7d	39.4 t	38.5 t	129.0 d
6	23.6 t	26.2 t	2.43 (brddd, 3.7, 9.0, 15.3) 3.06 (brddd, 6.9, 10.9, 15.3)	29.1 t	24.5 t	24.7 t	128.2 d
7	127.7 d	126.3 d	5.09 (brdd, 3.7, 10.9)	126.3 d	124.9 d	125.9 d	25.6 t
8	132.1 s	131.5 s		130.5 s	133.3 s	133.2 s	128.8 d
9	39.2 t	38.8 t	2.04 (m) 2.19 (m)	38.9 t	39.7 t	39.5 t	127.9 d
10	25.1 t	23.5 t	2.28 (m) 2.01 (m)	23.5 t	23.4 t	24.1 t	25.6 t
11	128.0 d	126.2 d	4.85 (brdd, 4.2, 7.4)	126.3 d	123.9 d	125.0 d	128.1 d
12	132.6 s	132.1 s		131.4 s	135.4 s	133.1 s	128.6 d
13	36.3 t	36.3 t	1.95 (dt, 6.7, 3.9) 2.08 (m)	36.4 t	39.8 t	37.5 t	25.6 t
14	25.2 t	24.9 t	1.80 (m) 2.07 (m)	26.3 t	28.9 t	28.2 t	127.5 d
15	75.1 s	72.3 s		149.7 s	74.6 s	73.4 s	130.5 d
16	29.5 q	27.3 q	1.19 (s)	108.8 t	29.9 q	27.6 q	27.3 t
17	22.2 q	26.9 q	1.13 (s)	21.5 q	24.6 q	27.6 q	29.3 t
18	24.2 q	19.9 q	1.80 (t, 1.5)	19.8 q	15.4 q	15.6 q	31.5 t
19	14.8 q	14.5 q	1.59 (brs)	14.4 q	15.2 q	15.3 q	22.6 t
20	15.0 q	14.3 q	1.53 (brs)	14.4 q	15.8 q	15.6 q	14.0 q

a Multiplicities determined by DEPT.
